# Liraglutide blocks the proliferation, migration and phenotypic switching of Homocysteine (Hcy)-induced vascular smooth muscle cells (VSMCs) by suppressing proprotein convertase subtilisin kexin9 (PCSK9)/ low-density lipoprotein receptor (LDLR)

**DOI:** 10.1080/21655979.2021.1982304

**Published:** 2021-10-19

**Authors:** Jingquan Ji, Ming Feng, Xiaohong Niu, Xinyu Zhang, Yilei Wang

**Affiliations:** a Department of Pathophysiology, Changzhi Medical College, Changzhi, Shanxi, China; b Department of Neurosurgery,Changzhi People’s Hospital, Changzhi, Shanxi, China; c Department of Endocrinology, The Heji Affiliated Hospital of Changzhi Medical College, Changzhi, Shanxi, China; d Department of Basic Medicine, Changzhi Medical College, Changzhi, Shanxi, China

**Keywords:** Liraglutide, PCSK9, LDLR, proliferation, migration

## Abstract

Liraglutide, a glucagon-like peptide 1 (GLP1) receptor agonist, is known to inhibit the atherosclerosis of apoE mice and suppress the cellular behaviors of VSMCs induced by AngII. This study aimed to explore whether liraglutide can reduce the proliferation, invasion and phenotypic transformation of VSMCs induced by Hcy and the underlying mechanism. Hcy was used to induce the proliferation of VSMCs, and liraglutide was then used to expose the cells for assessing cell proliferation. Afterward, the cell migration and phenotypic switch were evaluated to observe the effects of liraglutide. Meanwhile, the expression of PCSK9 and LDLR was detected. After overexpressing PCSK9, the changes in proliferation, cell migration and phenotypic switch were estimated again. Hcy promoted cell proliferation of VSMCs, whereas liraglutide blocked the proliferation, migration and phenotypic switch of Hcy-induced VSMCs. Furthermore, the expression of PCSK9 was downregulated and LDLR expression was upregulated after liraglutide administration in Hcy-induced VSMCs. After overexpressing PCSK9, the proliferation, migration and phenotypic switch of Hcy-induced VSMCs were enhanced. Liraglutide blocked the proliferation, migration and phenotypic switching of Hcy-induced VSMCs by suppressing PCSK9/LDLR. This finding provided the basis for the future application of liraglutide as an effective drug for therapeutic strategy in targeting AS.

## Introduction

Homocysteine (Hcy) a sulfur-containing amino acid, which is an important intermediate product in the metabolism of methionine and cysteine. Hyperhomocysteine is a risk factor of cardiovascular and cerebrovascular diseases, such as stroke, and a major risk factor for atherosclerosis (AS) and affects normal liver function [1–3]. Endothelial cell dysfunction and the phenotypic switching of vascular smooth muscle cells (VSMCs) are reported to involve in AS progression [4–8]. The recognition of differentially expressed genes in AS revealed potential dysregulated molecular pathways and genes involving in the pathogenesis of AS [9–12]. Glucagon-like peptide-1 (GLP-1), a member of incretin hormones along with gastric inhibitory peptide (GIP), is synthesized by L-cells in the distal small intestine and secreted in response to food intake [13]. GLP-1 analogues are potential anti-diabetic drugs which are considered to show favorable influence on bone metabolism in clinical practice [14]. It has been demonstrated that the beneficial effects of GLP-1 and GLP-1 agonists on the cardiovascular system do not rely on lowering blood glucose levels, but rather on improving endothelial dysfunction and left ventricular dysfunction.

Liraglutide is a clinical agent with an acylated GLP-1 analog that could improve glycemic control in patients with diabetes [14]. Immunohistochemical study showed that GLP1 receptor was expressed in VSMCs [15]. Liraglutide, a type of GLP1 receptor agonist, can inhibit the atherosclerosis of apoE mice and suppress the cellular behaviors of VSMCs induced by AngII [16]. Another studies have shown that liraglutide can attenuate advanced glycation end products-induced phenotypic transformation of coronary artery smooth muscle cells by inhibiting the expression of NF-κB in VSMCs [17], indicating that liraglutide has an obvious protective effect on VSMCs. However, no long-term studies on whether liraglutide can alleviate the protective effect of VSMCs induced by homocysteine (Hcy) are currently underway. Previous studies have shown that liraglutide can alleviate Alzheimer’s disease induced by Hcy [18]. We assume that liraglutide could prevent VSMCs from Hcy-induced cell injury. Therefore, this paper aims to explore whether liraglutide can reduce the proliferation, invasion and phenotypic transformation of VSMCs induced by Hcy and figure out the mechanism of liraglutide against Hcy.

## Materials and methods

### Cell culture and transfection

The human aortic VSMCs were obtained from American-type culture collection and cultured in DMEM (DMEM, Gibco, Grand Island, NY, USA) supplemented with 10% FBS, 100 U/ml penicillin, and 100 mg/ml streptomycin in a humidified incubator at an atmosphere of 37°C with 5% CO_2_. VSMCs were stimulated with Hcy and then treated with liraglutide (Novo Nordisk, Bagsværd, Denmark) at different concentrations (0.1, 1, and 10 μmol/L) for 24 h. For cell transfection, PCSK9 overexpression plasmids were transfected into VSMCs using Lipofectamine® 2000 reagent (Thermo Fisher Scientific, Inc., USA) and the effects of transfection were confirmed by further RT-qPCR analysis.

Quantitative Reverse-Transcription Polymerase Chain Reaction (qRT-PCR)

Total RNA was extracted from the cells using Trizol reagent (Invitrogen, Carlsbad, CA, USA) for mRNA analyses. First-strand cDNA was reverse transcribed from total RNA by the use of PrimeScript RT reagent (TaKaRa Biotech Corporation, Dalian, China). For the detection of mRNA expression, qRT-PCR was performed using Quantities SYBR Green PCR Kit (Invitrogen, CA, USA). All qRT-PCR experiments were conducted in triplicate for each condition. Relative fold changes in gene expression were calculated using the 2^−ΔΔCq^ method [19] with GAPDH as the internal control.

### Cell counting kit-8 (CCK-8) assay

The pre-treated cells were seeded in a 96-well plate (Corning, Inc.) at a density of 5 × 10^3^ cells/well. 10 μl CCK-8 (Sigma–Aldrich; Merck KGaA) solution was added into the wells for 4 h after the culture medium had been removed. The optical density value at a wavelength of 490 nm was read by a microplate reader (Thermo Fisher Scientific, Inc.).

### 5-ethynyl-2ʹ-deoxyuridine (EdU) staining

Treated VSMCs were cultured in a 96-well plate and fixed in 4% paraformaldehyde for 15 min at room temperature and permeabilized with 0.3% Triton X-100 for 15 min at room temperature. Next, the cells were washed thrice using PBS, and cultured with 0.5 mL of Click Reaction Mixture for 30 min in the dark. Finally, cell nucleus was counterstained with Hoechst (1:1000) for 10 min. The number of proliferative cells (EdU-positive) was counted under a fluorescence microscope (Olympus, Tokyo, Japan).

### Cell migration assay

Cells were plated in 96-well plates and cultured until a complete monolayer was formed. Then, a pipette tip was used to create a line on the monolayer of cells, and the location and images were recorded with the use of a light microscope (Olympus Corporation; magnification, ×200).

### Transwell assay

Tanswell assay was performed by referring to a previous study. Briefly, cells were grown into the upper chamber of 24-well transwells. After 24 h, the cells in the upper surface of the filter membranes were cleaned out and migrated cells were suffered from stain of 0.5% crystal violet and observed under a light microscope.

### Western blot analysis

Total protein was extracted from cells after transfection. Protein concentration was measured with bicinchoninic assay kit (Thermo Fisher Scientific, Inc.) according to the manufacturer’s protocol. An equal amount of protein (20 µg) was loaded to 12% SDS-PAGE gels (Bio-Rad Laboratories, Inc.) to separate the lysates. The proteins were then transferred onto polyvinylidene difluoride (PVDF) membranes (Invitrogen, CA, USA) and sealed with 5% skim milk for 1 h at room temperature. The membranes were then incubated with primary antibodies at 4°C overnight, following by an incubation with a secondary antibody at room temperature for 2–3 h. Gene expression was exhibited as a ratio of target gene level to GAPDH. Band visualization was conducted with the application of the electrochemiluminescence detection system (Invitrogen, CA, USA).

### Statistical analysis

All data were expressed as mean ± standard deviations (SD) of three independent experiments. A two-tailed Student’s t-test was used for the comparison of differences between two independent groups. One-way ANOVA was to compare the differences among multiple groups and post hoc Tukey’s test was conducted for between groups.

## Results

Liraglutide blocks the proliferation of Hcy-induced VSMCs

**Figure 1.**
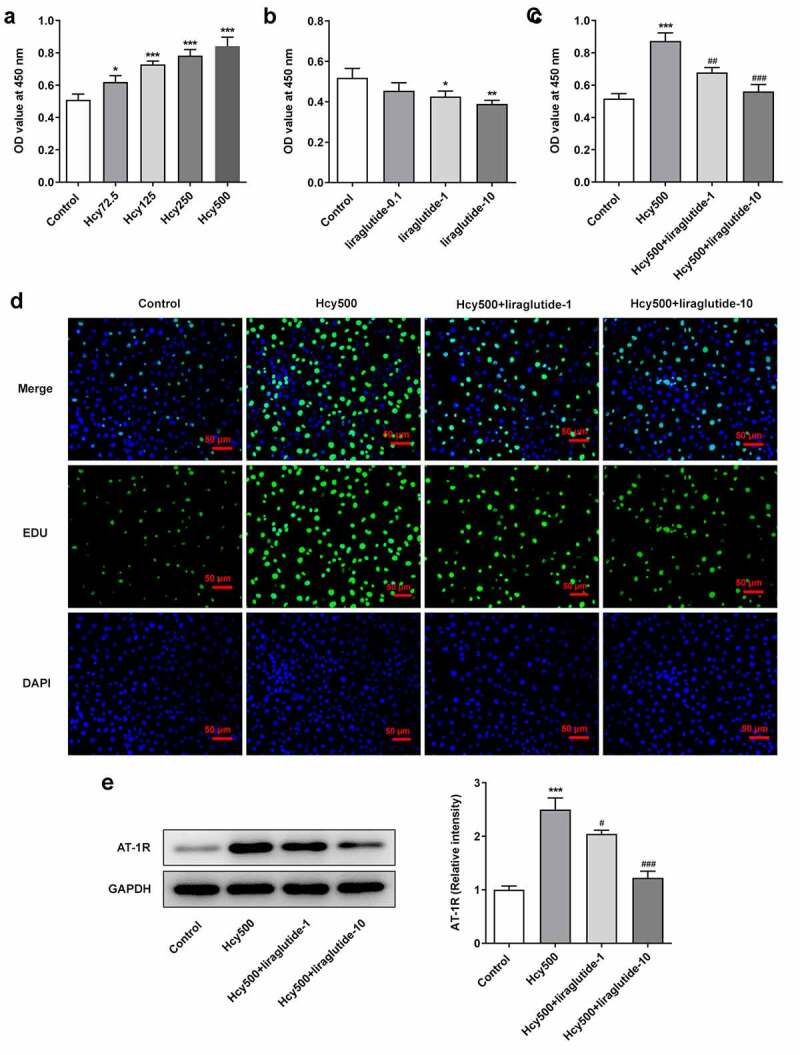
Liraglutide blocks the proliferation of Hcy-induced VSMCs. The OD values at 450 nm when (a) increasing doses of Hcy, (b) liraglutide and (c) Hcy and liraglutide were used on the VSMCs, detected by CCK-8 assay. (d) The images of TUNEL-positive cells. (e) The protein expression of AT-1 R in Hcy-induced VSMCs treated with liraglutide. *P < 0.05, **P < 0.01 and ***P < 0.001 vs. Control group; ^#^*P < *0.05, ^##^*P < *0.01 and ^###^*P < *0.001 vs. Hcy500 group

To measure the effect of liraglutide on AS, Hcy was used to induce VSMCs. With respect to the dosage of Hcy for optimal induction, results of CCK-8 detection indicated that the OD value increased following the treatment of elevated concentrations of Hcy in VSMCs (Figure 1(a)). For the convenience of observation, Hcy at a concentration of 500 μM was selected for the induction of VSMCs as it induced the highest level of proliferation, and liraglutide at the concentrations of 1 μmol/L and 10 μmol/L was used for the following treatment (Figure 1(b)). Additionally, CCK-8 and EDU analyses demonstrated that the proliferation of VSMCs was alleviated by liraglutide in Hcy-induced VSMCs (Figure 1(c,d)). As renin-angiotensin system (RAS) is closely associated with VSMCs, the Angiotensin Type 1 Receptor (AT-1 R), located in the brain, pituitary, adrenal gland, and kidney, plays an essential role in regulating the proliferation of VSMCs [20]. As suggested by western blot analysis, Hcy-induced high expression of AT-1 R was attenuated by liraglutide (Figure 1(e)). These results all imply that liraglutide blocks the proliferation of Hcy-induced VSMCs.

Liraglutide blocks the migration and phenotypic transition of Hcy-induced VSMCs

**Figure 2.**
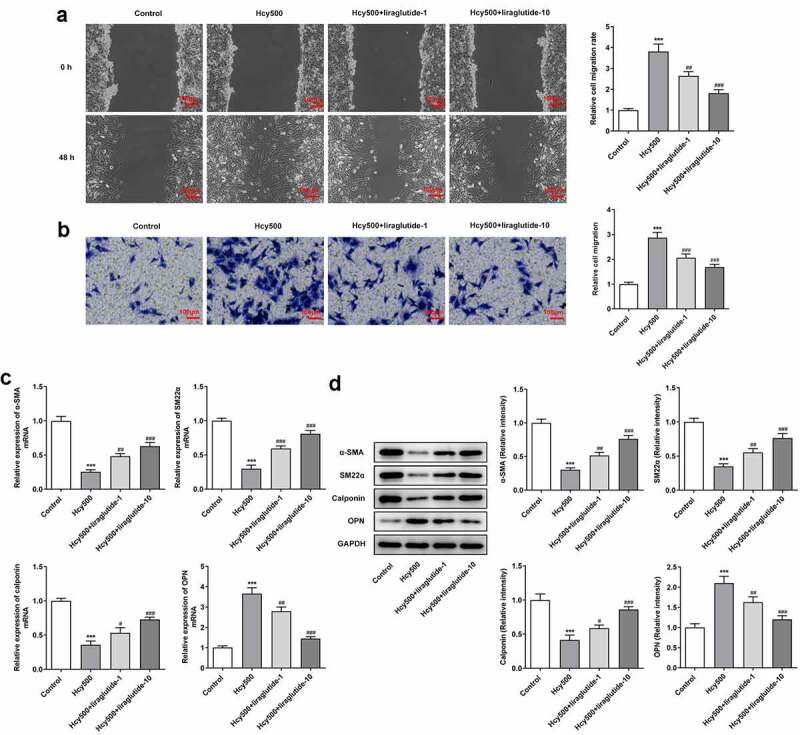
Liraglutide blocks the migration and phenotypic switching of Hcy-induced VSMCs. (a-b) The migration and (c-d) mRNA and protein expression of contractile proteins in Hcy-induced VSMCs treated with liraglutide. ***P < 0.001 vs. Control group; ^#^*P < *0.05, ^##^*P < *0.01 and ^###^*P < *0.001 vs. Hcy500 group

Due to the fact that cell migration is related to atherosclerosis, the migration ability of VSMCs was determined. The critical role of phenotypic transition has been defined by many studies, and suppressing phenotypic transition has been determined to be a feasible method for the treatment of AS [21]. Compared with Hcy 500 μm group, the width created by the pipette tip was enlarged in Hcy 500 μM+liraglutide 1 μmol/L group (Figure 2(a,b)). Meanwhile, the expression of differentiation-markers α-SMA, SM22α, and calponin suppressed by Hcy, and then was restored by liraglutide administration, while the expression of osteopontin (OPN) exhibited the opposite effect (Figure 2(c,d)). Taken together, liraglutide blocks the migration and phenotypic transition of Hcy-induced VSMCs.

Liraglutide alleviates the proliferation of Hcy-induced VSMCs by suppressing PCSK9/LDLR

**Figure 3.**
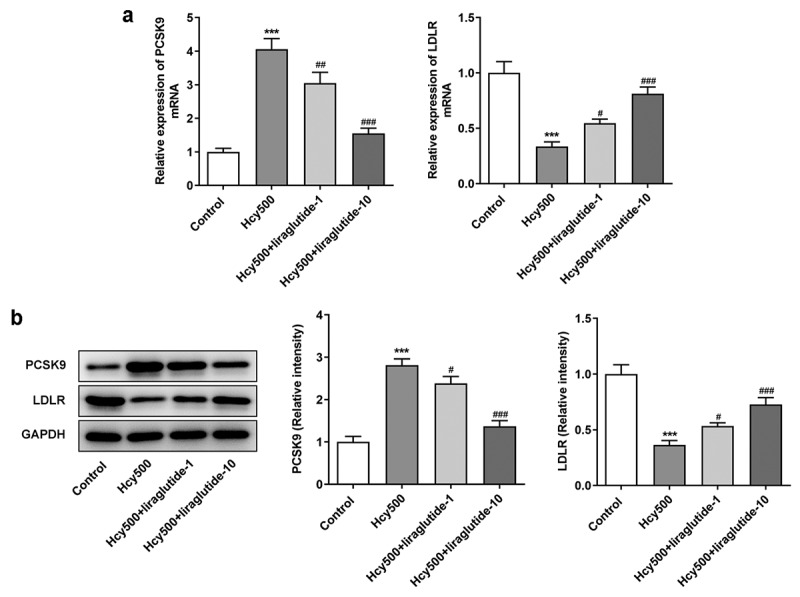
Liraglutide downregulates the expression of PCSK9 while upregulates that of LDLR in Hcy-induced VSMCs treated with liraglutide. (a-b) The mRNA and protein expression of PCSK9 and LDLR in Hcy-induced VSMCs treated with liraglutide. ***P < 0.001 vs. Control group; ^#^*P < *0.05, ^##^*P < *0.01 and ^###^*P < *0.001 vs. Hcy500 group

**Figure 4.**
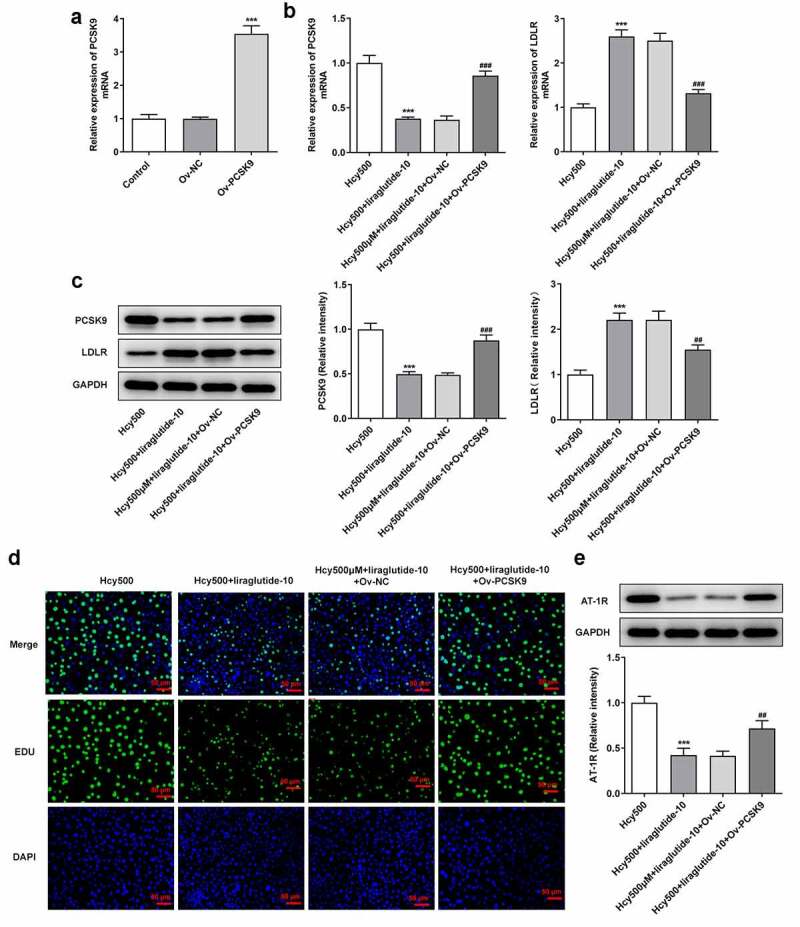
Liraglutide alleviates the proliferation of Hcy-induced VSMCs by suppressing PCSK9/LDLR. (a) The expression of PCSK9 after creating the overexpression plasmid of PCSK9. ***P < 0.001 vs. Control group. (b-c) The mRNA and protein expression of PCSK9 and LDLR in Hcy-induced VSMCs treated with liraglutide. ***P < 0.001 vs. Hcy 500 group; ^##^*P < *0.01 and ^###^*P < *0.001 vs. Hcy500+ liraglutide-10 group. (d) The apoptosis of Hcy-induced VSMCs co-treated with liraglutide and Ov-PCSK9 (the plasmids overexpressing PCSK9). (e) The expression of AT-1 R in Hcy-induced VSMCs co-treated with liraglutide and Ov-PCSK9. ***P < 0.001 vs. Hcy500 group; ^##^*P < *0.01 vs. Hcy500+ liraglutide-10 group

Recently investigations suggest that liraglutide can suppress PCSK9 expression in diabetes-influenced hepatocytes, and PCSK9 plays an important role in atherosclerosis [15,16]. Moreover, PCSK9 can bind to LDLR, the elevated expression of which is conducive to the progression of atherosclerosis [17]. Thus, the expression of PCSK9 and LDLR was measured in our study to validate the effects of liraglutide on Hcy-induced VSMCs. As shown in Figure 3(a,b), Hcy stimulated increased expression of PCSK9 in VSMCs, while suppressing LDLR expression. However, it was evident that liraglutide administration suppressed the expression of PCSK9 and increased LDLR expression. Subsequently, the plasmid overexpressing PCSK9 was created to increase its expression for the validation of the role PCSK9/LDLR in the proliferation of Hcy-induced VSMCs (Figure 4(a)). Notably, PCSK9 overexpression elevated the expression of PCSK9 but decreased that of LDLR in Hcy-induced VSMCs treated with liraglutide (Figure 4(b,c)). Additionally, results in Figure 4(d-f) suggested that the liraglutide-attenuated proliferation of Hcy-induced VSMCs was promoted by PCSK9 overexpression. Therefore, liraglutide alleviates the proliferation of Hcy-induced VSMCs by suppressing PCSK9/LDLR.

Liraglutide blocks the migration and phenotypic transition of Hcy-induced VSMCs by suppressing PCSK9/LDLR

**Figure 5.**
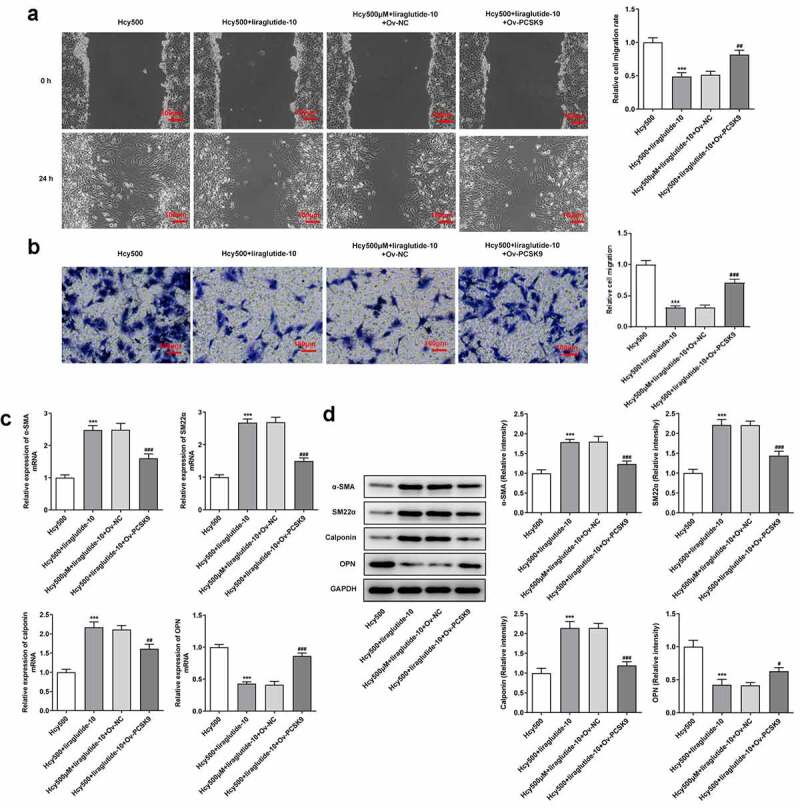
Liraglutide blocks the migration and phenotypic switching of Hcy-induced VSMCs by suppressing PCSK9/LDLR. (a-b) The migration and (c-d) mRNA and protein expression of contractile proteins in Hcy-induced VSMCs treated with liraglutide and Ov-PCSK9. ***P < 0.001 vs. Hcy500 group; ^#^*P < *0.05, ^##^*P < *0.01 and ^###^*P < *0.001 vs. Hcy500+ liraglutide-10 group

The underlying mechanism of liraglutide in the migration and phenotypic transition of Hcy-induced VSMCs was then investigated. In the presence of PCSK9, the migrated number of Hcy-induced VSMCs treated with liraglutide was elevated, reversely demonstrating that the migration of Hcy-induced VSMCs was inhibited by liraglutide via the suppression of PCSK9/LDLR expression (Figure 5(a,b)). As for molecules related to the phenotypic transition of VSMCs, the present data exhibited that PCSK9 overexpression decreased the levels of α-SMA, SM22α and calponin, while having an opposite effect on OPN level in Hcy-induced VSMCs treated with liraglutide (Figure 5(c,d)). Overall, liraglutide blocks the migration and phenotypic transition of Hcy-induced VSMCs by suppressing PCSK9/LDLR.

## Discussion

GLP-1 R agonists, which exploit the physiological impacts of GLP-1, assert its impacts by potently reducing glucose level and disposing part of the pathophysiological features of type 2 diabetes, a progressive and multifaceted disease [22]. As patients with type 2 diabetes are inclined to manifest with cardiovascular disease more often than non-type 2 diabetes patients, the ultimate goal of this study turned to investigate the potential efficacy of liraglutide in cardiovascular disease, including AS [23,24]. Previous outcome trials have also conferred cardiovascular benefits to GLP-1 R agonists [25]. The present study, to our best knowledge, is the first to associate and further verify the role of liraglutide, a type of GLP-1 R agonist, in AS by a series of functional assays.

Abnormal proliferation of VSMCs is a critical contributor to the pathophysiological course of AS [21]. VSMCs contribute to functional and structural changes in the arterial wall, the aggregation of which will trigger the occurrence of AS [26]. It is well known that Hcy is an independent risk factor for a wide range of cardiovascular diseases, including AS. Thus, this study introduced Hcy in VSMCs, which are predominant elements of the vascular media, to induce the pathological conditions of AS [27]. Of note, Hcy induced high proliferation of VSMCs, as suggested by the elevated OD values and EDU staining images following Hcy exposure. Consistent with a previous study showing that Exendin-4 (a GLP-1 R agonist) prevented the proliferation of VSMCs, this study also indicated the suppressive role of liraglutide in the VSMC proliferation [28].

The migration and phenotypic transition of VSMCs play a critical role in the progression of atherosclerosis [29]. Phenotypic transition is denoted by a lack of contractile proteins and rises in migration and proliferation [30]. In this study, we observed that Hcy-abated α-SMA, SM22α, and calponin expression was increased while Hcy-induced high expression of OPN was decreased by liraglutide, implying that liraglutide inhibited the phenotypic transition of Hcy-induced VSMCs. Additionally, we found that the decreased migration of VSMCs by liraglutide was reversed by the induction of PCSK9 overexpression. It has been reported that there existed a link between PCSK9 and endothelial cells migration. Hepatocyte nuclear factor 1α, which was associated with cell migration, could lead to increased expression of PCSK9 [31–33], which was considered to play a role in the influences of PCSK9 on the migration of VSMCs. As an essential protector for cholesterol homeostasis, PCSK9 is the latest member of the protein convertase family and leads to the elevation of LDL-c by affecting the level of LDLR [34]. Substantial experimental reports have elucidated the mechanisms where PCSK9 exerts regulatory influence in AS [35]. Previously indication that PCSK9 was upregulated in VSMCs upon the stimulus of TLR-4 ligand LPS was also validated in this study, except for the differences of stimulus used on the cells [36]. Interestingly, liraglutide use evidently decreased the levels of PCSK9 and increased its expression at the mRNA and protein levels bound to LDLR. PCSK9 inhibitors have been suggested to suppress the development of AS by influencing the level of circulating LDL-c [37]. By inducing the overexpression of PCSK9, LDR levels were markedly reduced when compared with the cotreatment group of Hcy and liraglutide. PCSK9 is able to destroy the LDL-R cell surface recycling and induce its degradation by its catalytic domain binding to the epidermal growth factor-A domain of the LDL-R [36,38,39]. Therefore, we guessed that liraglutide inhibited the proliferation and phenotypic switching of Hcy-induced VSMCs by blocking the PCSK9/LDLR signal.

As expected, the inhibitory role of liraglutide in the Hcy-induced VSMCs was abolished by overexpression of PCSK9, which consolidated our speculation.

## Limitation

The results of the study highlight the need for future research to detect the blood glucose level of patients with AS and further reveal the mechanism of liraglutide. There were studies reporting that Hcy-induced inflammation in vascular smooth muscle cells [40,41]. However, whether liraglutide affects inflammation in Hcy-induced VSMCs still requires deeper exploration.

## Conclusion

The present study focuses on the regulatory role of liraglutide in relation to the behaviors of Hcy-induced VSMCs. We have summarized that liraglutide blocks the proliferation, migration and phenotypic switching of Hcy-induced VSMCs by suppressing PCSK9/LDLR, the understanding of which underpins the future application of liraglutide as a potent drug for therapeutic strategy targeting AS.

## Highlights

1. Liraglutide plays anti-proliferative, invasion and phenotypic switching roles in Hcy-induced VSMCs.

2. Liraglutide suppressed VSMCs proliferation induced by Hcy via PCSK9.

3. Liraglutide inhibited VSMCs invasion and phenotypic switching caused by Hcy via PCSK9.
